# Geographical distribution of protozoan and metazoan parasites of farmed Nile tilapia *Oreochromis niloticus* (L.) (Perciformes: Cichlidae) in Yucatán, México

**DOI:** 10.1186/s13071-016-1332-9

**Published:** 2016-02-03

**Authors:** Amelia Paredes-Trujillo, Iván Velázquez-Abunader, Edgar Torres-Irineo, David Romero, Víctor Manuel Vidal-Martínez

**Affiliations:** Laboratorio de Parasitología, Centro de Investigación y de Estudios Avanzados del Instituto Politécnico Nacional, Unidad Mérida Km. 6 Carretera Antigua a Progreso, Cordemex, Mérida, Yucatán 97310 México; Departamento de Geografía, Universidad Nacional Autónoma de México, Circuito Interior, Ciudad Universitaria 04510, México, D.F México

**Keywords:** Nile tilapia farms, Parasites, Geographical distribution, *Gyrodactylus cichlidarum*, Management practices, Yucatán, México

## Abstract

**Background:**

In Yucatán State, southern México, as in many other parts of the world where tilapia has been introduced for aquaculture, the deficient application of management measures has led to the establishment of non-native parasites. The aims of this study were to describe the geographical distribution of protozoan and helminth parasites of farmed Nile tilapia *Oreochromis niloticus* (L.) throughout the Yucatán and to examine the potential statistical associations of the prevalence and mean abundance of these parasites with management and environmental variables.

**Methods:**

All 29 Nile tilapia farms currently operating in Yucatán were surveyed. Maps were created to describe the geographical location of the parasites infecting Nile tilapia at each farm. We evaluated the statistical associations of management and environmental variables with the mean abundance values of each parasite species using a multivariate redundancy analysis (RDA) and generalized additive models (GAM). We also used Ripley’s K to determine whether there were significant clusters of the mean abundance of particular parasite species in specific regions of the Yucatán State.

**Results:**

A total of 580 *O. niloticus* were examined, and 11 species of parasites were recorded. *Cichlidogyrus sclerosus* was the most frequent and abundant parasite at all 29 farms, whereas *Gyrodactylus cichlidarum* was found in 26 of the 29 farms. The RDA showed that the most important predictors were the concentration of nitrites and ammonium and the water exchange rate. The GAM showed the significant effect of the tank capacity, no use of quarantine area and no use of prophylactic treatments on the mean abundance of *G. cichlidarum*. The geographical distribution patterns of the mean abundance of most parasite species exhibited clustering near to the coast of Yucatán.

**Conclusion:**

Two groups of farms were distinguished: (i) farms with medium to high technology, where the most frequent and abundant parasite was *G. cichlidarum*, and (ii) farms with low technology, where the most frequent and abundant parasite was *C. sclerosus.* Good biosecurity practices such as the use of quarantine and prophylactic treatments prior to the introduction of infected Nile tilapia to the farms are recommended to avoid the establishment of parasites such as *G. cichlidarum* in farms.

**Electronic supplementary material:**

The online version of this article (doi:10.1186/s13071-016-1332-9) contains supplementary material, which is available to authorized users.

## Background

In the last two decades, tilapia has become one of the most commercially important freshwater fish species in global aquaculture [1, 2]. In the Americas, the increased production of farmed tilapia has been due to its adaptability to a diverse array of production systems, including extensive pond culture, semi-intensive cage culture, intensive flow-through tank culture, raceway culture and various highly intensive indoor methods [3]. In 2009, México produced 71,358 tons of tilapia and was the sixth largest producer of tilapia in the Americas after Brazil, Honduras, Colombia, Ecuador, and Costa Rica [1]. The production methods ranged from stocking fingerlings by release into reservoirs to intensive methods in ponds, lake cages, tanks, and shrimp ponds [3]. However, the intensification of crop densities in tilapia aquaculture, deficient management practices and a lack of biosecurity plans as defined by the World Organization for Animal Health (OIE) have led to the spread and establishment of non-native parasitic diseases [4, 5]. Some studies have identified poor management conditions such as stocking density, fish source, high concentrations of nitrates and low frequency of water exchange in cultured tilapia as being the factors associated with reports of parasitic (*Cichlidogyrus* spp., Coccidia, *Trichodina* sp., and *Gyrodactylus* sp.) and bacterial diseases [6–8]. Apart from deadly bacteria such as *Streptococcus iniae* Pier, 1976 [5, 8], the most common health problems in tilapia aquaculture are due to helminth parasites, especially monogeneans, which have produced economic losses attributed to slow growth, reduced fertility rates, and high mortality rates [9]. In Yucatán (a tropical state in southeastern México), the Nile tilapia [*Oreochromis niloticus* (L.)] culture is a rapidly growing commercial activity in rural areas. The monogeneans *Cichlidogyrus* spp. are the most frequent and abundant parasites in farmed Nile tilapia in Yucatán, yet their geographical distribution and the associated risk factors have not been previously reported [10].

Mapping techniques have been useful as descriptive analytical tools in numerous epidemiological studies, mainly regarding diseases that represent public health problems (e.g., leishmaniasis, schistosomiasis, and trypanosomiasis) [11]. These studies have described disease spread throughout different geographical areas, identified high-risk regions, and observed the natural historical variation of disease. These epidemiological maps have been useful as powerful monitoring tools in aquatic animal health for preventing geographical translocation of potential pathogens and determining the occurrence or distribution of exotic or endemic diseases, including changes in prevalence during different time periods [12]. Additionally, this approach could assist risk-based surveillance and help to monitor and predict the impact of environmental changes on the prevalence and severity of emerging endemic diseases (i.e. increasing in prevalence or range) [13, 14]. We hypothesise that the use of geographical tools such as maps, in combination with multivariate and nonlinear statistical analyses for determining meaningful environmental and management variables, will be valuable for adequate sanitation management in the Nile tilapia farms of Yucatán. Therefore, the aims of this study were to describe the geographical distribution of protozoan and helminth parasites of farmed Nile tilapia in Yucatán and to analyse the potential statistical associations between the prevalence and mean abundance of these parasites with management and environmental variables, with particular emphasis on *Gyrodactylus cichlidarum* Paperna, 1968, a well-known monogenean pathogen of the Nile tilapia.

## Methods

We conducted a census of all 29 Nile tilapia farms currently operating in Yucatán and registered with the Yucatán Aquatic Animal Health Committee (CESAY), which, in turn, graciously provided us with transportation and contact with the farm owners. To describe the parasite populations, we used measures of infection such as prevalence, mean abundance and mean intensity [15]. Prevalence was defined as the number of individuals of a host species infected with one or more particular parasite species, divided by the total number of hosts examined for that parasite species (expressed as a percentage) [15]. The abundance was defined as the total number of individuals of a particular parasite species in an individual from a particular host species [15]. The mean abundance was defined as the total number of individuals of a particular parasite species in a sample taken from a particular host species, divided by the total number of hosts of that species examined (including both infected and uninfected hosts) [15]. The mean intensity was defined as the average intensity of a particular species of parasite among the infected members of a particular host species [15].

We constructed a database with the information of the measures of infection of the ectoparasites (prevalence and mean abundance) of the 29 extant Nile tilapia farms in Yucatán. The information for the database was obtained from a census program undertaken by the senior author who visited the Nile tilapia farms during 2013 and from a historical database containing information regarding the measures of infection by parasites from all 29 Nile tilapia farms (2011 to 2012). This database was produced by the Aquatic Pathology Laboratory at CINVESTAV - Mérida, based on the parasitological analysis of Nile tilapia requested by CESAY. In both cases, the same measures of infection were used: prevalence and mean abundance of all the helminth and protozoan species infecting Nile tilapia at each farm. The sample size was 20 fish per farm, and the choice of this sample size was based on three considerations. Firstly, we assumed that the prevalence was 20 %, which was sufficient for us to collect species of ectoparasites with relatively high prevalence values, that is, the ones relevant in aquaculture [16]. Secondly, we accepted a probability α (alpha) that 5 % of all random samples drawn had no infected fish when the population was indeed infected. Thirdly, we assumed that the sensitivity of the sampling technique used (the stereomicroscope as a method for parasite detection) was 75 % due to the possibility of human error. We based this assumption on the fact that, even when the technicians at CINVESTAV are well trained in the parasitological search with the stereomicroscope, new personnel may be at risk of missing the parasites if occurring in very low numbers or in the very small larval stages. Because the values of sensitivity of microscopy as a diagnostic test for parasites of Nile tilapia have not been published, we assumed the level of sensitivity to be 75 %, which is similar to the one reported for the sensitivity of microscopy as a diagnostic test for malaria (77 %) [17].

With respect to specificity, we assumed a confidence level of 95 % due to the low probability for the misclassification of the parasites by our group, which has extensive experience in the taxonomy and identification of the monogeneans of cichlids [18, 19]. Thus, assuming a Poisson distribution for the probability of detecting species with at least 20 % of prevalence, the monthly sample size was obtained using the formula *n* = 4/prev, where *n* is the fish sample size; the number 4 originated from the formula derived from a Poisson distribution – Ln (α = 0 · 05(the accepted probability))/ 0.75 (sensitivity of the diagnostic method); and prev is the prevalence in the fish population (in this case, the assumed 20 %) [16]. Therefore, the formula *n* = 4/ prev, as *n* = 4/0.2 = 20 fish per farm was applied. At each farm, the fish were collected using a 2-m diameter cast net with a 2.5-cm mesh from a randomly selected production tank. We obtained the temperature (°C), conductivity (μS/cm), dissolved oxygen (mg/l), salinity (ppt), nitrites (mg/l), nitrates (mg/l) and ammonium (mg/l) levels using a multiparameter meter (YSI-85) [20]. The live fish were transported in containers with artificial aeration to the Aquatic Pathology Laboratory at CINVESTAV - Mérida. Once in the laboratory, each individual Nile tilapia was kept in a container with freshwater and an oxygen supply and was euthanized with a 100 mg/L of benzocaine until opercular movements ceased; the brain was severed posteriorly by spiking. Immediately afterward, each fish was measured to obtain the total length (TL, cm), standard length (SL, cm) and total weight (W, g).

Skin, gills, scales from the lateral line and fins were examined under a stereomicroscope for ectoparasites. For endoparasites, the organs were separated individually into Petri dishes with 0.07 % saline solution. Liver, kidney, brain, spleen, heart, muscle, mesentery and intestine were reviewed by compression between two 10 cm squared pieces of glass. Once the parasites were found, they were counted, preliminarily identified and fixed depending on the taxonomic group [18]. The monogeneans were removed with paintbrushes, stained with ammonium picrate and identified according to suitable literature (e.g. [18, 21]). The protozoans were stained with silver nitrate and identified [22].

To display the geographical location of each Nile tilapia farm and that of the parasites infecting Nile tilapia at each farm in Yucatán, maps were created with the geographical coordinates of the farms by using the *mapplots* package [23] in the statistical software R, version 2.9.1. To determine whether the spatial distribution of the mean abundance of each parasite species was spatially random or clustered, Ripley’s *K* (r) function was used under a homogeneous Poisson assumption [24]. This function is flexible because it takes into account all possible pairs of points and not merely the nearest pairs [25]. We plotted the Ripley´s *K* function *vs* the radius of complete spatial randomness to assess if the parasites species presented spatial clusters [25]. If the observed Ripley´s *K* function was above the theoretical Ripley´s *K* function, then the point pattern was not random (i.e. clustered). If a point fulfilled the assumption of non-randomness, then mean density (= mean abundance) was estimated using an isotropic Gaussian kernel smoothing. For n points, the shape of a kernel smoothing estimator may be expressed as follows [24, 25]:

$$ \widehat{\lambda}(x)=\frac{1}{h^2}{\displaystyle \sum_{t=1}^n\kappa \left(\frac{\left\Vert x-{x}_i\right\Vert }{h}\right)}/q\left(\left\Vert x\right\Vert \right) $$

where *κ* (*u*) is a bivariate symmetrical kernel function, *q* (||*x*||) represents a border correction to compensate for missing observations that occur when *x* is close to the border region, and the bandwidth *h* measures the level of smoothing. In this step, the analysis was made using an R implementation for analysing spatial point patterns in the package *spatstat* [26].

A total of 39 management and environmental variables related to the Nile tilapia production cycle, including the education level of the farmers, were collected from 21 of the 29 farms (Table 3). To obtain information on the management procedures at each production unit, a standardised questionnaire was designed and applied following the method described [27] (Additional file 1). The management variables were then semi-quantitatively categorised according to [27]. From the 29 farms, only 21 owners were willing to answer the questionnaire. The farms were then classified based on (i) the mean annual production, number of workers, and nearby communities; (ii) the biosecurity measures including disinfection of equipment, tank cleaning, and vector presence; (iii) the presence of facilities/management such as quarantine area, bathroom, footbaths, warehouse, guardhouse; and (iv) an established monitoring procedure to collect information on environmental variables such as nitrates, nitrites, salinity and temperature (Table 3).

We performed a redundancy analysis (RDA) using the Canonical Correspondence Analysis software CANOCO to determine whether there were statistical associations between management and environmental variables and the abundance values of each parasite species per individual fish. RDA is a direct ordination analysis that identifies compositional gradients in a biological data set (e.g. a parasite population or community) as a response to measured environmental factors (in this case, environmental factors obtained from each Nile tilapia farm) [28].

Redundancy analysis was used because the lengths of the ordination axes were less than two standard deviations [29]. In this case, it is assumed that most of the response curves for the number of individuals of the metazoan and protozoan parasite species, with respect to the environmental variables, will be linear. If the lengths of the ordination axes are longer than two standard deviations, then unimodal models, such as canonical correspondence analysis (CCA), should be used [29, 30].

RDA is a direct extension of regression analysis to model multivariate response for dependent variables (in this case, the abundance values of each parasite species) regressed on several explanatory independent variables with linear constraints on the regression coefficients [28, 29]. Since RDA is similar to a multivariate linear regression, linearity is an important requirement. Variables with large deviations from linearity were log-transformed, and the improvement in linearity re-checked. The significance of all statistical analyses was established at an α = 0.05 unless otherwise stated. To determine the statistical significance (*p* < 0.05) of the canonical axes for mean abundance of parasites with respect to the environmental and management variables, a Monte Carlo simulation based on 4,999 permutations was used [28, 30].

We used a generalized additive model of location, shape and scale (GAMLSS), which includes nonparametric and nonlinear smoothing functions [31], to determine the potential effect of the independent management and environmental variables on the mean abundance of *Gyrodactylus cichlidarum* (the dependent variable). The GAMLSS allows flexibility when specifying the frequency distribution of the response variable such as Gaussian, negative binomial or Poisson distributions, among 39 other available specific distributions (31). This algorithm also allows us to model all distribution parameters as functions of independent variables. The model allows modelling not only of the mean (or location) but also of other parameters of the distribution of the dependent variable such as the linear and/or nonlinear, parametric and/or additive nonparametric and functions of independent variables and/or random effects. Hence, GAMLSS is especially suited for modelling a dependent variable that does not follow an exponential family distribution or when the scale or shape of the frequency distribution of this dependent variable changes with the independent variables [31]. In this case, we chose the Poisson distribution because the value of the Akaike information criterion (AIC) was the lowest when compared with different frequency distributions (e.g. negative binomial or Gaussian distributions) [32]. The GAMLSS was fitted assuming a Poisson error distribution because the dependent variable (mean abundance of *Gyrodactylus cichlidarum*) consisted of counts and the $\eta$ =ln ($\overline{y}$) function was used, where $\overline{y}$ is the dependent variable and $\eta$ is the link function [31, 32]. The GAMLSS model also included data for sites where *G. cichlidarum* was absent, and the best model was selected based on the lowest AIC, lowest global deviance value and highest percentage of explained deviance [32]. A stepwise model selection was applied to select the independent variables. The GAM was fitted using regression cubic splines (*cs*) for the smooth terms regarding only these continuous variables. The GAM was fitted using the GAMLSS package of the R software [33].

## Results

A total of 580 Nile tilapia from all 29 farms in Yucatán were examined, and 11 species were recorded. The values of prevalence and mean abundance of the monogenean and protozoan species for each farm are presented in Table 1. The monogeneans were represented by nine species, followed by the protozoans with two species (Table 1). *Cichlidogyrus sclerosus* Paperna & Thurston, 1969 and *Cichlidogyrus tilapiae* Paperna, 1960 were the most frequent and abundant monogeneans at every farm (Table 1). Fish in all farms were infected with *C. sclerosus*, with an overall prevalence of 74 % (confidence intervals (CI) of 70–77 %) and a mean abundance of 73.83 ± 134.29 parasites per fish (Table 1). The pathogenic monogenean *Gyrodactylus cichlidarum* was found at 26 of 29 farms visited, with an overall prevalence of 31 %. Table 2 presents the values for prevalence, mean abundance, and mean intensity of each monogenean and protozoan species for the number of fish examined in each of the 29 extant farms in Yucatán.

**Table 1:** Species composition and measures of infection of the ectoparasites (protozoans and helminths) of *Oreochromis niloticus* (L.) for the 29 extant farms in Yucatán, México. The calculation of these measures of infection was based on the 580 fish examined across all 29 tilapia farms

Parasite species	Infection site	Estimated prevalence^a^ (%)	Abundance	Intensity	Intensity	Intensity
		(CI-95 %)	Mean ± SD	Mean ± SD	Median	Range
PROTOZOA						
*Trichodina* sp.	Skin	41 (36–45)	12.04 ± 49.95	29.3 ± 74.68	10	1–980
*Vorticella* sp.	Skin	8 (5–8)	1.22 ± 7.20	14.88 ± 20.89	6	1–82
MONOGENEA						
*Cichlidogyrus sclerosus* Paperna & Thurston, 1969	Gills	74 (70–77)	73.83 ± 134.29	99.25 ± 147.51	46	1–11178
*Cichlidogyrus tilapiae* Paperna, 1960	Gills	65 (61–70)	32.35 ± 74.24	51.83 ± 91.52	28	1–885
*Cichlidogyrus dossoui* Paperna, 1960	Gills	44 (39–48)	8.64 ± 22.82	19.51 ± 31.06	12	1–356
*Cichlidogyrus longicornis* Paperna & Thurston, 1969	Gills	39 (33–42)	6.36 ± 20.71	16.37 ± 30.69	8	1–246
*Cichlidogyrus quaestio* Douëllou, 1993	Gills	2 (1–1)	1 ± 0.26	1.7 ± 0.94	2	1–4
*Cichlidogyrus* sp.	Gills	42 (37–46)	15.98 ± 47.99	38.01 ± 68.19	14	1–746
*Cichlidogyrus halli* (Price & Kirk, 1967)	Gills	4 (1–4)	1 ± 3.64	14.6 ± 12.57	7	1–44
*Gyrodactylus cichlidarum* Paperna, 1968	Fins, skin	31 (26–33)	2.00 ± 8.67	6.49 ± 14.86	9	1–173
*Enterogyrus malmbergi* Bilong Bilong, 1988	Intestine	13 (10–15)	1.06 ± 9.42	8.02 ± 24.93	3	1–169

^a^Expected apparent prevalence following [16]

*Abbreviations*: *CI* Confidence intervals, *SD* Standard deviation

**Table 2:** Measures of infections with protozoan and helminth ectoparasites of *Oreochromis niloticus* (L.) (n = 20) for the 29 extant tilapia farms in Yucatán, México

Farms (n = 29)	Measures of infection	Cs	Ct	Cd	Cl	Cq	Ci	Ch	Gy	Tr	Vo	En
	P	70 (65-74)	95 (90-99)	65 (59-69)	40 (35-47)	0	55 (51-54)	0	40 (35-47)	40 (35-47)	0	10 (6-15)
1	MA	14.1 ± 22.03	34.8 ± 38.92	9.45 ± 14.73	14.8 ± 54.65	0	16.95 ± 43.28	0	1.95 ± 3.17	12.5 ± 24.84	0	0.15 ± 0.48
	MI	20.14 ± 24.05	49.71 ± 39.09	13.5 ± 16.24	21.14 ± 76.28	0	24.21 ± 55.58	0	2.78 ± 3.31	17.85 ± 31.72	0	0.21 ± 0.70
	P	70 (65-74)	50 (45-56)	55 (50-60)	35 (32-40)	0	20 (16-26)	0	15 (11-20)	35 (32-40)	5 (1-8)	20 (16-26)
2	MA	41.5 ± 54.26	4.3 ± 6.49	9.4 ± 10.44	2.1 ± 3.21	0	1.3 ± 2.77	0	0.45 ± 1.23	3.3 ± 5.69	0.1 ± 0.44	0.25 ± 0.55
	MI	59.28 ± 56.28	6.14 ± 6.93	13.42 ± 7.91	3 ± 2.30	0	1.85 ± 1.91	0	0.64 ± 1.73	4.71 ± 5.94	0.14 ± 0.44	0.35 ± 0.5
	P	85 (79-89)	85 (79-89)	20 (16-26)	20 (16-26)	20 (16-26)	50 (45-56)	0	20 (16-26)	65 (59-69)	10 (6-15)	15 (11-20)
3	MA	153.3 ± 174	115.9 ± 182	5.6 ± 16.57	1.65 ± 5.18	0.25 ± 0.55	24.7 ± 43.66	0	0.65 ± 1.59	6.95 ± 8.84	0.15 ± 0.48	1.05 ± 3.80
	MI	180.35 ± 176	136.35 ± 190	28 ± 30.06	8.25 ± 9.87	1.25 ± 0.54	49.4 ± 51.66	0	3.25 ± 2.21	10.69 ± 9.07	1.5 ± 1	7 ± 8.66
	P	80 (76-88)	40 (35-47)	50 (45-56)	50 (45-56)	10 (6-15)	35 (32-40)	5 (1-8)	15 (11-20)	50 (45-56)	5 (1-8)	10 (6-15)
4	MA	52.4 ± 97.80	10.5 ± 19.33	5.95 ± 9.09	5.9 ± 8.42	0.1 ± 0.30	8.4 ± 18.30	0.2 ± 0.89	0.3 ± 0.80	4.9 ± 8.69	0.75 ± 3.35	0.1 ± 0.30
	MI	65.5 ± 105	26.25 ± 23.28	11.9 ± 9.80	11.8 ± 8.50	1 ± 0	24 ± 24.97	4 ± 0.89	2 ± 1	9.8 ± 10.31	15 ± 3.35	1 ± 0
	P	100 -	55 (51-54)	30 (26-36)	75 (70-80)	0	50 (45-56)	0	64 (59-68)	85 (79-89)	35 (32-40)	35 (32-40)
5	MA	151 ± 216.46	47.25 ± 113.89	4.2 ± 7.89	8.65 ± 8.58	0	28.2 ± 45.87	0	3.5 ± 3.69	35.3 ± 60.38	2.55 ± 5.22	0.75 ± 1.64
	MI	161 ± 216.46	85.90 ± 144.88	14 ± 8.39	11.53 ± 8.02	0	56.4 ± 51.72	0	5.38 ± 3.24	41.52 ± 63.68	7.28 ± 6.79	2.14 ± 0.89
	P	95 (90-99)	55 (50-60)	50 (45-56)	35 (32-40)	0	40 (35-47)	15 (11-20)	5 (1-8)	55 (50-60)	5 (1-8)	0
6	MA	38.8 ± 34.30	8.4 ± 12.14	9.65 ± 19.50	3.8 ± 6.51	0	24.25 ± 76.05	2.8 ± 8.21	0.05 ± 0.22	20.35 ± 53.98	0.1 ± 0.44	0
	MI	40.84 ± 33.97	15.27 ± 12.84	19.3 ± 24.42	10.85 ± 6.71	0	60.62 ± 114	18.66 ± 14.04	1 ± 0	37 ± 69.70	2 ± 0	0
	P	80 (76-88)	55 (50-60)	55 (50-60)	45 (40-51)	0	45 (40-51)	5 (1-8)	35 (32-40)	55 (50-60)	5 (1-8)	0
7	MA	66.05 ± 94.31	11.7 ± 13.72	3.8 ± 7.19	3.05 ± 4.71	0	11 ± 20.19	0.4 ± 1.78	1.05 ± 2.23	68.7 ± 217	0.05 ± 0.22	0
	MI	82.56 ± 99.06	21.27 ± 11.56	6.90 ± 8.64	6.77 ± 4.94	0	24.44 ± 24.46	8 ± 8	3 ± 3	124.90 ± 286	1 ± 1	0
	P	80 (76-88)	80 (76-88)	40 (35-47)	55 (50-60)	0	55 (50-60)	10 (6-15)	35 (32-40)	65 (59-69)	0	25 (20-29)
8	MA	87.5 ± 221	20.35 ± 34.05	4.75 ± 8.28	4.8 ± 6.56	0	15.9 ± 24.31	1.9 ± 6.85	1.75 ± 3.05	14.2 ± 26.03	0	0.45 ± 0.88
	MI	109.37 ± 244	25.43 ± 36.48	11.87 ± 9.47	8.72 ± 6.64	0	28.90 ± 26.64	19 ± 15.55	5 ± 3.26	21.84 ± 29.87	0	1.8 ± 0.83
	P	95 (90-99)	45 (40-51)	65 (59-69)	55 (50-60)	0	60 (54-66)	0	60 (54-66)	85 (79-89)	25 (21-29)	25 (20-29)
9	MA	81.05 ± 143	10.95 ± 22.34	12.8 ± 17.46	22.35 ± 34.10	0	6.9 ± 8.51	0	1.7 ± 1.86	36.45 ± 35.23	13 ± 25.34	1.3 ± 2.73
	MI	85.31 ± 146	24.33 ± 28.61	19.69 ± 18.32	40.63 ± 37.31	0	11.5 ± 8.21	0	2.83 ± 1.58	42.88 ± 34.36	52 ± 22.71	5.2 ± 3.19
	P	100 -	50 (45-56)	75 (69-80)	75 (69-80)	0	65 (59-69)	15 (11-20)	40 (35-47)	10 (6-15)	10 (6-15)	15 (11-20)
10	MA	189.1 ± 187	63.6 ± 152	19.7 ± 32.34	10.8 ± 10.92	0	54.15 ± 166	2.2 ± 7.64	4.1 ± 8.29	0.65 ± 2.30	0.55 ± 2.03	0.4 ± 1.09
	MI	190.1 ± 187	127.21 ± 199	26.26 ± 35.14	14.4 ± 10.31	0	83.30 ± 203	14.66 ± 16.77	10.25 ± 10.71	6.5 ± 4.94	5.5 ± 4.94	2.66 ± 1.52
	P	95 (90-99)	75 (69-80)	70 (65-74)	65 (59-69)	0	65 (59-69)	0	30 (26-36)	5 (1-8)	10 (6-15)	5 (1-8)
11	MA	111.6 ± 121	52.5 ± 68.55	27.7 ± 46.99	9.8 ± 16.29	0	19.9 ± 38.04	0	2.1 ± 4.21	0.1 ± 0.44	1.4 ± 6.02	0.1 ± 0.44
	MI	117.47 ± 122	70 ± 71.16	39.57 ± 52.17	15.07 ± 18.28	0	30.61 ± 44.01	0	7 ± 5.13	2 ± 2	14 ± 18.38	2 ± 2
	P	60 (54-66)	50 (45-56)	35 (32-40)	35 (32-40)	0	20 (16-26)	0	10 (6-15)	20 (16-26)	20 (16-26)	0
12	MA	12.55 ± 23.26	17.25 ± 39.95	3.6 ± 7.80	4.45 ± 10.59	0	4.2 ± 10.02	0	0.3 ± 0.97	1.25 ± 2.84	1.4 ± 3.66	0
	MI	20.91 ± 27.28	34.5 ± 52.05	10.28 ± 10.60	12.71 ± 15.26	0	21 ± 12.90	0	3 ± 1.41	6.25 ± 3.09	7 ± 5.71	0
	P	20 (16-26)	45 (40-51)	5 (1-8)	10 (6-15)	0	10 (6-15)	0	15 (11-20)	5 (1-8)	45 (40-51)	0
13	MA	3.2 ± 9.34	4.8 ± 11.83	0.1 ± 0.44	0.3 ± 0.97	0	0.4 ± 1.23	0	0.7 ± 2.10	0.1 ± 0.44	4.4 ± 8.18	0
	MI	16 ± 16.73	10.66 ± 16.18	2 ± 2	3 ± 1.41	0	4 ± 0	0	4.66 ± 3.78	2 ± 2	9.77 ± 9.99	0
	P	100 -	70 (65-74)	85 (79-89)	55 (50-60)	0	80 (76-88)	0	70 (65-74)	70 (65-74)	5 (1-8)	15 (11-20)
14	MA	195.2 ± 247	79.5 ± 130	42.3 ± 76.69	20.2 ± 51.85	0	59 ± 76.07	0	3.3 ± 4.25	7.65 ± 11.54	0.4 ± 1.78	1.6 ± 4.76
	MI	198.20 ± 246	113.57 ± 144	49.76 ± 81.18	36.72 ± 66.64	0	73.75 ± 78.54	0	4.71 ± 4.39	10.92 ± 12.49	8 ± 8	10.66 ± 8.38
	P	95 (90-99)	90 (86-96)	60 (54-66)	50 (45-56)	0	70 (65-74)	0	40 (35-47)	45 (40-51)	0	10 (6-15)
15	MA	112.4 ± 51.73	82.35 ± 84.85	13.4 ± 20.11	10.9 ± 17.05	0	48.35 ± 60.81	0	1.35 ± 1.87	4.05 ± 6.55	0	0.25 ± 0.71
	MI	118.31 ± 45.67	91.5 ± 84.62	22.33 ± 21.93	21.8 ± 18.70	0	69.07 ± 62.15	0	3.37 ± 1.30	9 ± 7.21	0	2.5 ± 1.15
	P	85 (54-66)	35 (32-40)	30 (26-36)	50 (45-56)	0	25 (21-29)	15 (11-20)	35 (32-40)	70 (65-74)	5 (1-8)	5 (1-8)
16	MA	93.4 ± 145	18.55 ± 40.59	3.2 ± 8.46	7.3 ± 10.75	0	8 ± 22.05	0.85 ± 2.80	3 ± 8.73	5.45 ± 7.10	0.15 ± 0.68	0.05 ± 0.22
	MI	109.88 ± 104	53 ± 53.60	10.66 ± 12.81	14.6 ± 10.91	0	32 ± 35.29	5.66 ± 5.50	8.57 ± 13.22	7.78 ± 7.25	3 ± 3	1 ± 1
	P	100 -	60 (54-66)	35 (32-40)	30 (26-36)	0	55 (50-60)	5 (1-8)	10 (6-15)	0	0	0
17	MA	153.25 ± 118	47.9 ± 62.67	7.1 ± 11.58	3.65 ± 8.48	0	28.95 ± 36.67	0.05 ± 0.22	0.15 ± 0.48	0	0	0
	MI	158.25 ± 120	79.83 ± 63.26	20.28 ± 10.62	12.16 ± 12.20	0	52.63 ± 36.15	1 ± 1	1.5 ± 0.70	0	0	0
	P	70 (65-74)	85 (54-66)	80 (76-88)	40 (35-47)	0	55 (50-60)	0	30 (26-36)	50 (45-56)	0	15 (11-20)
18	MA	12.15 ± 11.24	30.4 ± 52.74	11.4 ± 11.21	2.2 ± 3.98	0	3.6 ± 5.28	0	1 ± 2.15	4.7 ± 8.20	0	0.25 ± 0.71
	MI	17.35 ± 9.34	35.76 ± 55.67	14.25 ± 10.76	5.5 ± 4.72	0	6.54 ± 5.64	0	3.33 ± 2.87	9.4 ± 9.64	0	1.66 ± 1.15
	P	90 (86-96)	85 (54-66)	65 (59-69)	40 (35-47)	0	75 (69-80)	0	10 (6-15)	15 (11-20)	0	15 (11-20)
19	MA	34.35 ± 22.52	18.75 ± 14.71	8.7 ± 14.18	10.05 ± 28.94	0	6.9 ± 8.96	0	0.2 ± 0.61	0.25 ± 0.63	0	0.25 ± 0.71
	MI	38.16 ± 20.31	22.05 ± 13.39	13.38 ± 15.62	25.12 ± 40.99	0	9.2 ± 9.29	0	2 ± 0	1.66 ± 0.57	0	1.66 ± 1.15
	P	95 (90-99)	80 (76-88)	70 (65-74)	70 (65-74)	0	65 (59-69)	20 (16-26)	55 (50-60)	35 (32-40)	10 (6-15)	60 (55-66)
20	MA	101.1 ± 144	18.5 ± 19.37	19 ± 19.54	8.4 ± 9.38	0	28.45 ± 47.53	5.3 ± 11.62	2.9 ± 6.17	14.55 ± 29.46	0.1 ± 0.30	20.35 ± 44.17
	MI	106.42 ± 146	23.12 ± 19.32	27.14 ± 18.69	12 ± 9.34	0	43.76 ± 54.54	26.5 ± 11.93	5.27 ± 7.86	41.57 ± 39.35	1 ± 0	33.91 ± 53.37
	P	5 (1-8)	80 (76-88)	15 (11-20)	20 (16-26)	15 (11-20)	0	0	50 (45-56)	55 (50-60)	0	5 (1-9)
21	MA	0.1 ± 0.43	9.4 ± 8.45	1.4 ± 4.01	2.2 ± 5.77	0.4 ± 1.02	0	0	3.95 ± 6.51	21.9 ± 36.76	0	0.05 ± 0.21
	MI	2 ± 2	11.75 ± 7.75	9.33 ± 7.02	11 ± 9.59	2.66 ± 1.15	0	0	7.9 ± 7.60	39.81 ± 43.27	0	1 ± 1
	P	40 (35-47)	60 (54-66)	0	7 (4-11)	0	13 (9-18)	7 (4-11)	5 (1-8)	33 (29-39)	20 (16-26)	0
22	MA	68.93 ± 165	19.26 ± 44.91	0	13.33 ± 51.63	0	0.93 ± 2.71	0.8 ± 3.09	2.4 ± 4.25	4.93 ± 9.83	6.53 ± 20.09	0
	MI	73.85 ± 171	32.11 ± 55.36	0	200 ± 200	0	7 ± 4.24	12 ± 12	4.5 ± 5.04	14.8 ± 12.47	32.66 ± 39.3	0
	P	64 (61-69)	43 (39-49)	0	0	0	0	0	0	14 (11-19)	0	0
23	MA	76 ± 112	4.71 ± 7.25	0	0	0	0	0	0.07 ± 0.26	0.28 ± 0.82	0	0.07 ± 0.26
	MI	118.22 ± 122	11 ± 7.34	0	0	0	0	0	0	2 ± 1.41	0	1 ± 1
	P	60 (54-66)	20 (16-26)	13 (9-18)	20 (16-26)	0	0	0	30 (26-36)	60 (54-66)	0	0
24	MA	3.33 ± 4.13	0.8 ± 1.69	0.26 ± 0.70	0.46 ± 0.99	0	0	0	20.6 ± 44.96	47.73 ± 55.84	0	0
	MI	5.55 ± 4.00	4 ± 1	2 ± 0	2.33 ± 0.57	0	0	0	38.62 ± 56.99	79.55 ± 51.09	0	0
	P	20 (16-26)	30 (26-36)	15 (11-20)	25 (21-29)	0	20 (16-26)	0	20 (16-26)	10 (6-15)	5 (1-8)	35 (31-40)
25	MA	0.65 ± 1.53	0.5 ± 1	0.15 ± 0.36	0.35 ± 0.67	0	3.6 ± 12.96	0	0.65 ± 2.03	0.5 ± 1.67	2.7 ± 12.07	0.65 ± 1.38
	MI	3.25 ± 1.89	1.66 ± 1.21	1 ± 0	1.4 ± 0.54	0	18 ± 26.82	0	3.25 ± 3.86	5 ± 2.82	54 ± 0	1.85 ± 1.86
	P	80 (76-88)	100 -	50 (45-56)	30 (26-36)	10 (6-15)	70 (65-74)	0	40 (35-47)	60 (54-66)	0	0
26	MA	210.4 ± 240	161 ± 161	8.6 ± 9.47	2.4 ± 5.64	0.2 ± 0.63	28.2 ± 28.30	0	2.1 ± 3.03	31.6 ± 39.86	0	0
	MI	263 ± 241	161 ± 161	17.2 ± 4.14	8 ± 8.71	2 ± 0	40.28 ± 25.17	0	5.25 ± 2.36	52.66 ± 39.09	0	0
	P	50 (45-56)	80 (76-88)	60 (54-66)	30 (26-36)	0 0	50 (45-56)	0	0	80 (76-88)	10 (6-15)	20 (16-26)
27	MA	47.2 ± 64.86	24 ± 21.41	3 ± 3.91	1.8 ± 3.32	0	7.6 ± 14.13	0	0	5.4 ± 6.94	0.1 ± 0.31	0.2 ± 0.42
	MI	94.4 ± 62.42	30 ± 19.59	5 ± 3.94	6 ± 3.46	0	15.2 ± 17.46	0	0	6.75 ± 7.18	1 ± 0	1 ± 0
	P	100 -	100 -	77 (71-83)	11 (7-16)	0	77 (71-83)	11 (7-16)	22 (19-26)	0	0	33 (28-37)
28	MA	62.88 ± 41.14	21.33 ± 16.30	4 ± 3	0.22 ± 0.66	0	7.77 ± 7.03	0.66 ± 2	0.22 ± 0.44	0	0	0.77 ± 1.39
	MI	60.88 ± 41.14	25.33 ± 16.30	5.14 ± 2.26	2 ± 0	0	10 ± 4.9	6 ± 0	1 ± 0	0	0	2.33 ± 1.52
	P	80 (76-88)	100 -	40 (35-47)	20 (16-26)	0	20 (16-26)	0	0	80 (76-88)	0	0
29	MA	2.2 ± 2.28	40.8 ± 24.35	0.6 ± 0.89	0.4 ± 0.89	0	10 ± 22.36	0	0	1.2 ± 1.09	0	0
	MI	2.75 ± 2.21	40.8 ± 24.35	1.5 ± 1	2 ± 0	0	50 ± 0	0	0	1.5 ± 1	0	0

*Abbreviations*: Cs, *Cichlidogyrus sclerosus*; Ct, *Cichlidogyrus tilapiae*; Cd, *Cichlidogyrus dossoui*; Cl, *Cichlidogyrus longicornis*; Cq, *Cichlidogyrus quaestio*; Ci, *Cichlidogyrus* sp.; Ch, *Cichlidogyrus halli*; Gy, *Gyrodactylus cichlidarum*; Tr, *Trichodina* sp.; Vo, *Vorticella* sp.; En, *Enterogyrus malmbergi*

Prevalence = P (CI-95 %), Mean abundance (± SD) = MA, Mean intensity (± SD) = MI, CI = Confidence intervals, SD = Standard deviation

*Expected apparent prevalence following [16]

### Geographical distribution of the parasites found in Nile tilapia throughout farms

**Fig. 1:**
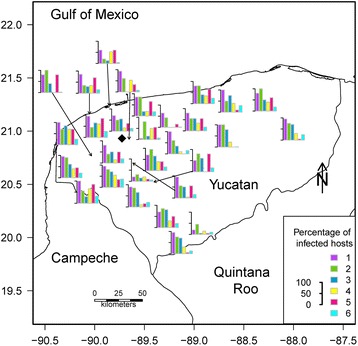
Map of the percentage of infected Nile tilapia *Oreochromis niloticus* (L.) from 29 farms in Yucatán, Southern México. The bar chart represents the percentage of fish infected with parasites in each tilapia farm. Each bar corresponds to a parasite species. The bigger the bar, the higher the percentage of infected hosts by each parasite species. The parasites are represented by a number as follows: 1, *Cichlidogyrus sclerosus*; 2, *Cichlidogyrus tilapiae*; 3, *Cichlidogyrus dossoui*; 4, *Gyrodactylus cichlidarum*; 5, *Trichodina* sp.; 6, other parasites (*Cichlidogyrus longicornis*, *Cichlidogyrus quaestio*, *Cichlidogyrus* sp., *Cichlidogyrus halli*, *Vorticella* sp., *Enterogyrus malmbergi*). ♦ = Mérida City

**Fig. 2:**
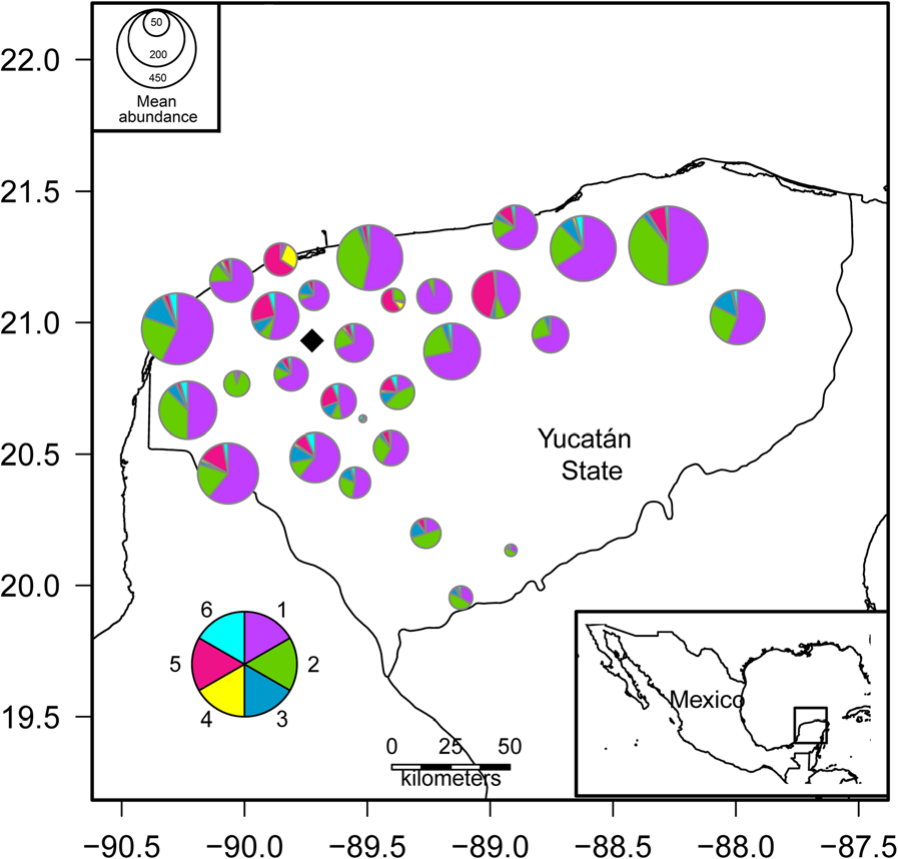
Map of mean abundance of the parasite species infecting the Nile tilapia *Oreochromis niloticus* (L.) from 29 farms in Yucatán, Southern México. The radius of each pie chart represents the total number of individual parasites of all species found in a Nile tilapia farm. Each slice corresponds to a parasite species. The bigger the slice, the higher the mean abundance of each parasite species. The parasites are represented by a number as follows: 1, *Cichlidogyrus sclerosus*; 2, *Cichlidogyrus tilapiae*; 3, *Cichlidogyrus dossoui*; 4, *Gyrodactylus cichlidarum*; 5, *Trichodina* sp.; 6, other parasites (*Cichlidogyrus longicornis*, *Cichlidogyrus quaestio*, *Cichlidogyrus* sp., *Cichlidogyrus halli*, *Vorticella* sp., *Enterogyrus malmbergi*). ♦= Mérida City

The 29 operating Nile tilapia farms were roughly located along the northwestern coast and southwestern edge of Yucatán State. The map of the percentage of infected hosts, including the most prevalent parasite species, is shown in Fig. 1. For each bar chart (=farm), the larger the bar, the higher the percentage of infected hosts for each parasite species. One of the most important parasites was *C. sclerosus* (indicated by the bar number one in the chart in the map), with a prevalence between 80 and 100 % per farm (Table 2). The map showing the mean abundance of parasites in farmed Nile tilapia is presented in Fig. 2. In this map, the larger the radius of the pie chart, the higher the total number of parasites was at each farm and the larger the slice, the higher the mean abundance of parasites. The most abundant parasite species were the monogeneans *C. sclerosus* and *C. tilapiae*, which, in most farms, represented 50 % or more of the individual helminths collected (Table 2). Moreover, the greatest number of individual helminths was collected from farms along the coast and in northwestern Yucatán State. *Gyrodactylus cichlidarum*, a very important parasite due to its pathogenicity, was present in 26 of the 29 extant Nile tilapia farms in Yucatán, with a prevalence ranging between 5 % (in farms 5 and 21) and 70 % (in farm 13), and with a mean abundance ranging from 0.05 ± 0.22 (in farm 6) to 20.60 ± 44.96 (in farm 24) (Table 2). The names of the farms were not provided by request of the farm owners.

### Clustered patterns of the parasitic helminths and protozoans of Nile tilapia in Yucatán

**Fig. 3:**
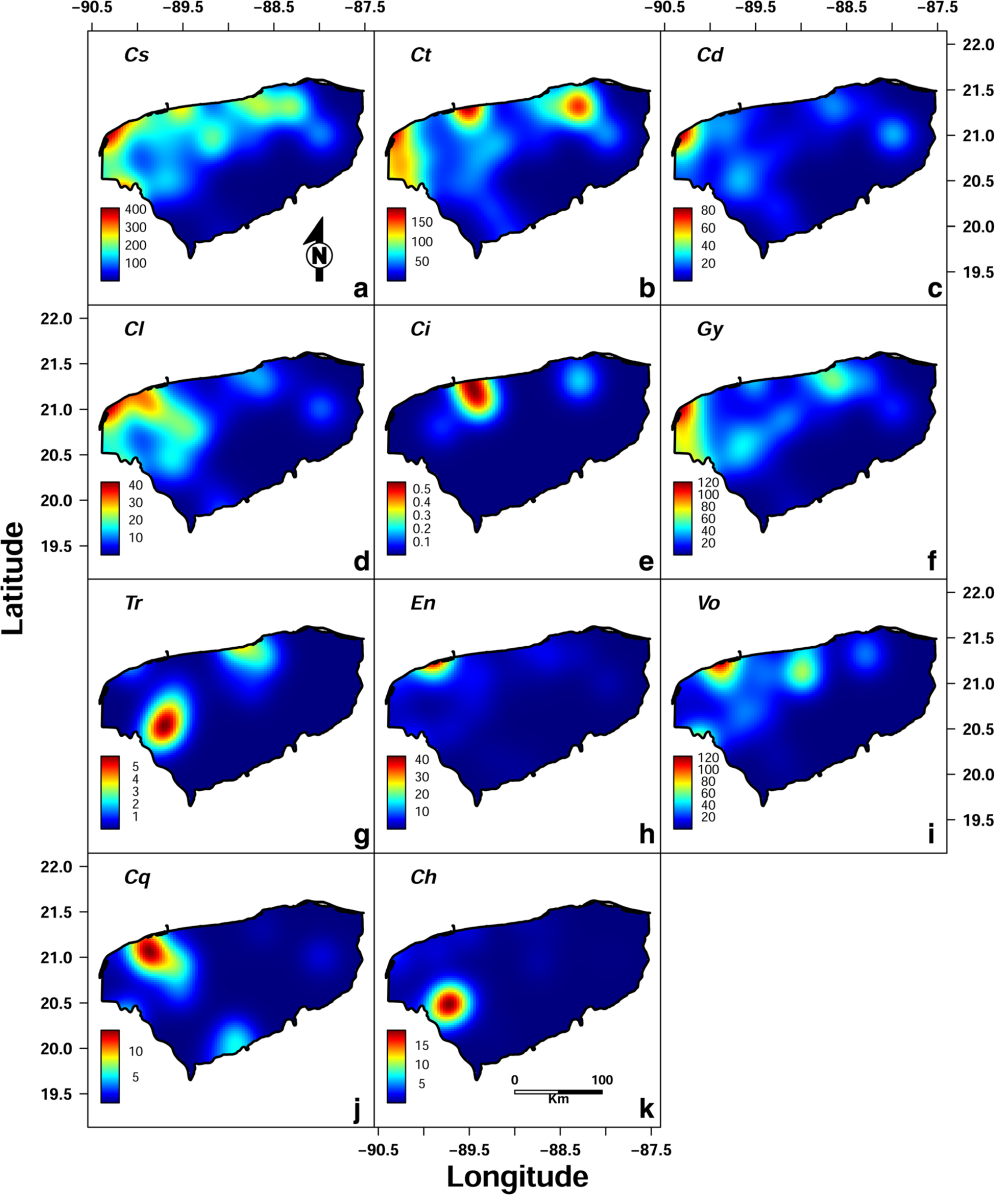
Spatial clustering of the density (mean abundance) of the ectoparasite species infecting the Nile tilapia *Oreochromis niloticus* (L.) in Yucatán, Southern México. Red coloured cluster zones indicate higher mean density of the parasite species. The lighter toned cluster zones indicate lower mean density of the parasite species. The parasites are represented by acronyms as follows: **a** Cs, *Cichlidogyrus sclerosus*; **b** Ct, *Cichlidogyrus tilapiae*; **c** Cd, *Cichlidogyrus dossoui*; **d** Cl, *Cichlidogyrus longicornis*; **e** Ci, *Cichlidogyrus* sp.; **f** Gy, *Gyrodactylus cichlidarum*; **g** Tr, *Trichodina* sp.; **h** En, *Enterogyrus malmbergi*; **i** Vo, *Vorticella* sp.; **j** Cq, *Cichlidogyrus quaestio*; **k** Ch, *Cichlidogyrus halli*

The geographical distribution pattern for the mean density (= mean abundance) of the parasite species showed a clustered pattern (see Additional file 2). The red clusters in the maps of Fig. 3 present a high mean density for each one of the parasite species collected from Nile tilapia in this study, and the blue zones are related to low mean density of these parasites. Most of the clusters of parasites were found near the coast, mainly in northwestern Yucatán, where most farms are located. The highest concentration of *C. sclerosus* was located in the northwest area of Yucatán (Fig. 3a). The highest mean density for *G. cichlidarum* depicted a similar spatial distribution pattern to that of *C. sclerosus* but with lower values (Fig. 3f).

### The questionnaire and the redundancy analysis

The results of the questionnaire applied to the farm owners (Table 3) indicated the presence of two groups of Nile tilapia farms in Yucatán. The first group had low technology with only a small number of concrete tanks (<4), low annual production (200–500 kg) with sub-standard sanitary measures, a lack of biosecurity knowledge, and represented 65 % of the total farmers interviewed. The second group included medium- to high-technology farms with better facilities including a higher numbers of tanks (>5), quarantine areas, guardhouses, better biosecurity measures in place and high annual production (1,000–25,000 kg). This group represented 35 % of the total farmers interviewed.

**Table 3:** List of environmental variables and management practices obtained from Nile tilapia production units between 2012 and 2013 in Yucatán State

Management practices (n = 32)	Low technology farms	Medium to high technology farms
Environmental variables (7)		
Fish age (months)	2–4.5	2.5–6.0
Mean annual production (kg)	200–500	1,000–25,000
Water supply	<1 km	>1 km
Number of tanks on the farm	1–4	5–13
Tank capacity (m^3^)	4–6.5	12–16
Fish density per tank	950–4,500	5,000–10,000
Number of workers	1–2	4–9
Nearby communities	<1 km	>1 km
Fingerlings source	Campeche	Campeche – Tabasco
Type of tanks	Concrete – geomembrane	Concrete – geomembrane
Epidemiological outbreaks	Parasites, bacteria, fungi	Parasites, bacteria
Prophylactic treatments	No	Salt, Crustabay
Quarantine area	No	Yes
Vector presence	pigs, cattle, poultry, dogs and cats	Sometimes dogs
Guardhouse	No	Yes
Footbaths	No use	Yes
Tank cleaning	No disinfection	Virkon, chlorine
Bathroom	Latrine / No use	Bathroom
Disinfection of equipment	No disinfection	Virkon, chlorine
Warehouse	Galley / Lacking	Cement
Drug use	No	Antibiotics
Incineration of organisms	No	Burying and incineration
Education level of workers	No education	Elementary school, sometimes preparatory
Drainage	No drainage	With drainage
Communication	Roadless	With road
Livestock areas	Poultry and pigs	Poultry
Agricultural areas	Yes	Yes
Accumulation of garbage	Yes	No
Accumulation of dead animals	Yes	Sometimes
Water exchange rate	Monthly and fortnightly	Weekly and fortnightly
Water discharge treatment	No	Sometimes
Other land uses	Husbandry and livestock	Sometimes livestock
Nitrates (mg/l)	4.21–7.57	0.10–5.0
Nitrites (mg/l)	0.002–2.97	1.83–3.10
Ammonium (mg/l)	1.85–3.38	0.01–1.68
Salinity (ppt)	0.30–0.70	0.83–2.11
Dissolved oxygen (mg/l)	1.98–7.68	7.78–13.61
Conductivity (μS/cm)	1044–1698	1831–4364
Temperature (T °C)	23.10–31	28.92–32

**Fig. 4:**
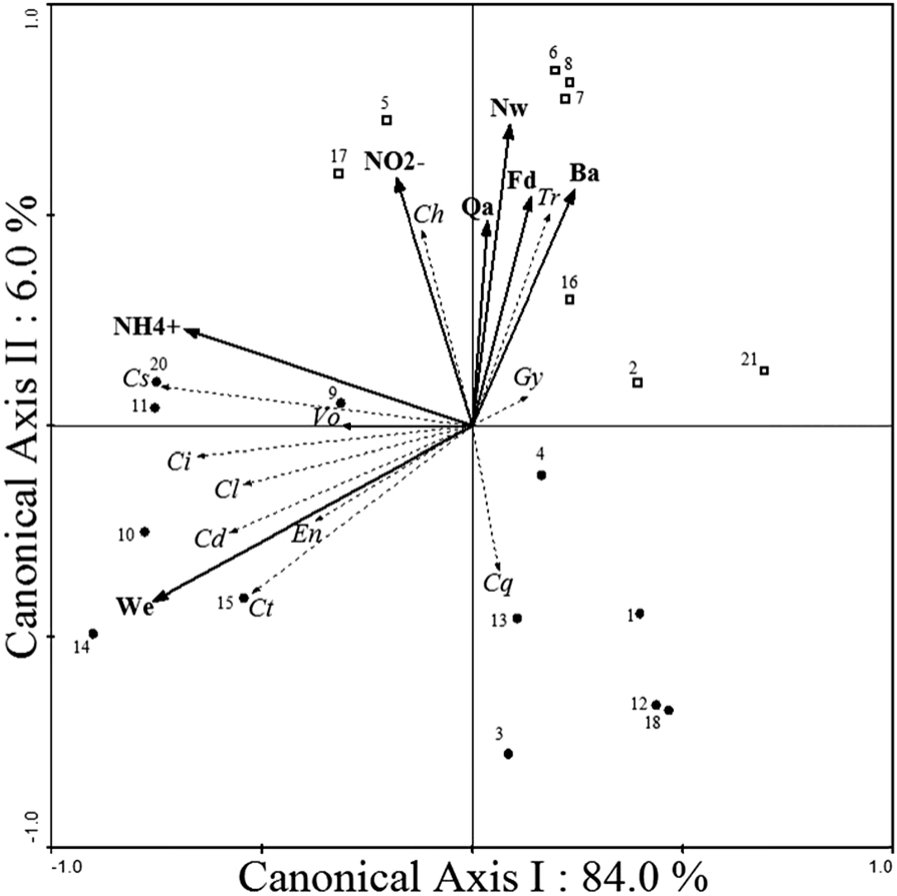
Redundancy analyses (RDA) showing the statistical association between the number of ectoparasite species of the Nile tilapia *Oreochromis niloticus* (L.) and the environmental and management variables of the farms. The acronyms for the parasite species were as follows: Cs, *Cichlidogyrus sclerosus*; Ct, *Cichlidogyrus tilapiae*; Cd, *Cichlidogyrus dossoui*; Cl, *Cichlidogyrus longicornis*; Cq, *Cichlidogyrus quaestio*; Ci, *Cichlidogyrus* sp.; Ch, *Cichlidogyrus halli*; Gy, *Gyrodactylus cichlidarum*; Tr, *Trichodina* sp.; Vo, *Vorticella* sp.; En, *Enterogyrus malmbergi*. Codes for management and environmental variables (in bold) are as follows: Fd, Fish density per tank; Nw, Number of workers; Qa, Quarantine; NO_**2**_^**−**^, Nitrites; Ba, Bathrooms; NH_**4**_^**+**^, Ammonium; We, Water exchange. The numbers associated with the circles and squares are the farm reference numbers in Table 3

The results of the redundancy analysis (RDA) using the abundance of each parasite species in each individual host as a dependent variable concur with those obtained by the questionnaire, splitting the farms into two groups with different types of ectoparasites. The RDA accounted for 90 % of the total variance and was significant for the first axis (F = 13.84; *P* = 0.04; 4,999 permutations) and all canonical axes (F = 2.65; *P* = 0.03; 4,999 permutations) (Fig. 4). The first group of farms had medium to high technology and production (farms labelled with diamonds in Fig. 4), where the fish density per tank, number of workers, the presence of a quarantine area, bathrooms, and the concentration of nitrites (in black arrows) were positively associated with the abundance of *G. cichlidarum*, *Trichodina* sp. and *Cichlidogyrus halli* (in dashed arrows) (Fig. 4). Due to its pathogenicity, a further analysis of *G. cichlidarum* was undertaken using general additive models to determine the potential statistical relations between the independent management and environmental variables at the farms and the abundance of this parasite species. The second group (farms labeled with filled circles) included farms with low technology, which showed positive associations between the abundance of several species of *Cichlidogyrus* and *Vorticella* sp. and two management variables (ammonium and water exchange rate) (Fig. 4).

### Statistical relations between the management and environmental variables of Nile tilapia farms and the mean abundance of *Gyrodactylus cichlidarum*

A total of 39 environmental and management variables (Table 3) were originally included in the GAMLSS model. However, after the stepwise procedure, only four independent variables were selected for the final GAMLSS model: one environmental (dissolved oxygen) and three management variables (tank capacity, use of quarantine area and use of prophylactic treatments). The model including these four variables showed 63 % deviance and had the lowest values of global deviance (GD = 48.05) and of the Akaike information criterion (AIC = 74.05; Table 4). Additional file 3 shows the nonlinear relations of each of the management and environmental independent variables with respect to the dependent variable (*G. cichlidarum* mean abundance). In regard to treatments, in Additional file 3, there were four treatments: formalin, salt, organophosphorus, and no treatment. However, only the last one (no treatment) was selected by the stepwise procedure.

**Table 4:** Generalized additive model of location, shape and scale (GAMLSS) fits for mean abundance of *Gyrodactylus cichlidarum* (MAG; dependent variable) in Nile tilapia farms in Yucatán state. The best model was selected using a stepwise procedure and the lowest values of Akaike information criterion (AIC) and global deviance. The final model was: MAG ~ *cs*(Do) + *cs*(Tc) + Qa + Pt where *cs* is a cubic spline smooth function, and those independent variables without smoother had a linear relationship with the dependent variable (MAG)

Model	Degrees of freedom (df)	Global deviance	Deviance explained (%)	AIC	*P* (0.05)
(Null model) MAG ~ 1	-	130.82	-	132.82	5.4e^-6^*
MAG ~ *cs*(Do)	df =16	100.52	23.16	110.51	0.0002*
MAG ~ *cs*(Do) + *cs*(Tc)	df =16	92.65	29.17	110.65	0.0059*
MAG ~ *cs*(Do) + *cs*(Tc) + Qa	df =19	86.29	34.03	106.29	0.0208*
MAG ~ *cs*(Do) + *cs*(Tc) + Qa + Pt	df =17	48.05	63.27	74.05	0.0001*

*Abbreviations*: *Do* Dissolved oxygen (mg/l), *Tc* Tank capacity (m^3^), *Qa* Use of quarantine area, *Pt* Use of prophylactic treatments

*P value < 0.05

## Discussion

The results showed a clustered pattern for the geographical distribution of the abundance for most of the parasite species of the Nile tilapia in Yucatán. *Cichlidogyrus sclerosus*, the most frequent and abundant monogenean species, showed a clustered pattern with the highest mean density (= mean abundance) in the northwestern corner of the state. A similar pattern was observed for the other species of *Cichlidogyrus* (*C. tilapiae*, *Cichlidogyrus dossui*, and *Cichlidogyrus longicornis*), and for *G. cichlidarum* (Fig. 3). This result, together with the RDA multivariate analysis, suggested that the high- and low-technology Nile tilapia farms in Yucatán have specific suites of ectoparasites, depending on their management and environmental variables. Thus, in medium- to high-technology Nile tilapia farms, *G. cichlidarum*, *Trichodina* sp., and *C. halli* were more prevalent and abundant, whereas the low-technology farms had more *C. sclerosus*, *C. tilapiae*, *C. dossoui*, *C. longicornis*, *Cichlidogyrus quaestio*, *Cichlidogyrus* sp., *Vorticella* sp. and *Enterogyrus malmbergi*.

### Parasite species composition of Nile tilapia farms

The monogenean and protozoan parasites found in *O. niloticus* from the farms (Table 1) have previously been reported in Yucatán State [9]. In the specific case of the monogeneans of the genus *Cichlidogyrus*, they have also been recorded in farms and wild crops of tilapia throughout different regions of México and the world [6, 7, 9, 10, 18]. The important point here was that these ectoparasites from the Yucatán aquaculture facilities were reported in relatively high numbers (Table 2). This is important because the few extant reports on the histopathology produced by *Cichlidogyrus* spp. in *O. niloticus* indicate that even at low numbers, these monogeneans can cause slight gill disease with hyperplasia, edema and epithelial sloughing [34]. Furthermore, the attachment structures (anchors) and cutaneous disorders (epithelial hyperplasia and proliferation of mucoid cells) can be an entry point for bacterial and fungal infections [34]. Although the present paper did not include a histopathological study, we consider that under the present circumstances of high prevalence and mean abundance of *Cichlidogyrus* spp. in most of the farms studied (Table 2), a histopathological study is necessary. Due to their direct life-cycles and attachment organs (hooks), these ectoparasites are likely to affect the intensive culture of Nile tilapia, particularly in combination with substandard management practices, high fish densities and favorable ecological conditions (e.g. high temperature) with regard to fish development [9]. Vidal-Martínez et al. [18, 19] have reported several species of the genus *Cichlidogyrus* in *Oreochromis aureus* (Steindachner) and *O. niloticus* from three southeastern Mexican states (Tabasco, Veracruz and Yucatán), suggesting that under deficient management conditions, these monogeneans can enhance parasite-induced host mortality [18]. Massive invasions of monogeneans of the genus *Cichlidogyrus* have also been reported in tilapia in Cuba, Colombia, the Philippines and Brazil [9].

*Gyrodactylus cichlidarum* was reported for the first time in Yucatán by [35] but only in one locality (Mérida). Consequently, all 26 farms in which this species has been reported in the present paper are new geographical records. *Gyrodactylus cichlidarum* infected up to 31 % of the Nile tilapia in these farms, whereas the protozoans *Trichodina* sp. and *Vorticella* had prevalences of 8 % and 41 %, respectively (Table 1). The high fish densities and substandard management practices in several Nile tilapia farms likely facilitate constant reinfection with these ectoparasites, which could be leading to host mortality. The presence of *Gyrodactylus* is important for Nile tilapia farming in Yucatán due to the pathogenicity of the species of this genus in other fish species [19, 35]. Only a single study has reported a fairly weak case of histopathology due to *G. cichlidarum* related to the mortality of fingerlings of farmed Nile tilapia in Peru [36]. However, Martins et al. [37] recently reported that tilapia vaccinated for *Streptococcus iniae* but parasitised with *Trichodina heterodentata* Duncan, 1977 and *G. cichlidarum* had significantly lower antibody levels and survival rates than tilapia that were not parasitised. Clearly, more histopathological and physiological studies on the response of tilapia to these species of ectoparasites are urgently needed.

### Geographical distribution of the parasites of Nile tilapia among farms in Yucatán

Most of the sampled Nile tilapia farms were located on the northwestern border of the coast and in the southeastern Yucatán State. The farms located in the coastal zone and along the northwestern border had higher levels of infection than the farms located in southeastern Yucatán. This could be due to the higher fish densities managed by these farms and greater commercial interaction among the farms in comparison with those from other regions of the state. Nabeil-Salama & Murray [38] found that the rate of infection by pathogens in the smaller, more separated fish farms decreased, whereas in larger, nearby farms, the spread of infection throughout the individuals was almost instantaneous. However, within these medium- to high-technology farms, the parasites persist but are clearly not considered to be pathogenic by the owners. We believe that this assumption is a mistake because these parasites can become harmful under the challenging environmental circumstances typical of fish farms (e.g. high temperature and productivity, low water exchange rate, and high fish density) [39]. Mardones et al. [40] found that the geographical dispersion of the salmon anemia virus in Chile is linked to anthropogenic activities. Thus, the movement of live or harvested fish or their byproducts was suspected to have played a more important role than environment or passive transmission. We observed fluctuations in the levels of infection of *C. sclerosus* and *C. tilapiae*, which could be due to risk management practices that influence the life-cycles of these parasites. Most Nile tilapia farms in Yucatán have substandard health conditions such as poor filter cleaning, lack of disinfection of equipment and tanks, and poor water quality, which in turn could favour constant parasitic reinfection. Akoll et al. [41] found that frequent seining and pond flushing can interrupt *C. sclerosus* and *C. tilapiae* transmission by washing out the oncomiracidia. Several authors have suggested that the transport of live fish, high stocking density, low frequency of pond drainage and a lack of disinfection of canoes and angling equipment are some of the most relevant management practices that enhance the transmission of ectoparasites in farms [42, 43].

### Clustered patterns of the parasitic helminths and protozoans of Nile tilapia in Yucatán

The estimated density of the clusters was consistent with the known abundance values of the parasite species in Nile tilapia farms (Table 2; Figs. 1 and 2). The most likely explanation for the high values of both estimated and actual abundance of these parasites in the farms were the high fish densities and deficient sanitation management, as well as increased anthropogenic and commercial interaction favouring parasite spread among farms throughout the coastal area of Yucatán. In fact, one of the main risk factors enhancing the dispersion and establishment of ectoparasites in the farms in Yucatán is likely to be the hatcheries producing infected Nile tilapia fingerlings and selling them without appropriate sanitary measures. Even when considering the difference between the discrete nature of the Nile tilapia farms based on the mainland Yucatán and those in open systems such as the Norway fjords, there are still some important similarities. The farms with high commercial interaction play a critical role in the spread of pathogenic infections in Norway [44]. In fact, fish farms located near (<5 km) to a harvest station have often been identified as a risk factor for disease transmission [44]. Harvest stations can be a source of infection to adjacent farms *via* other pathways such as fomites (e.g. fishing equipment and vehicles) [45, 46]. Clearly, there is a large need for the application of biosecurity measures such as those defined by the World Organization for Animal Health (OIE) [4] to diminish, as much as possible, the spread and establishment of the ectoparasites of Nile tilapia among farms in Yucatán.

### The questionnaire and statistical associations between management and environmental variables for specific types of parasites in the Nile tilapia farms

The results of the questionnaire applied at each Nile tilapia farm indicated that most workers and owners have very basic levels of education (elementary school = 6 years at most), and many of them are illiterate, often lacking technical training in aquaculture (Table 3). These people also have a poor understanding of fish health management, which is reflected in the deficient management conditions strongly associated with high nitrite concentration levels and specific types of ectoparasites for both types of farms in Yucatán. The multivariate redundancy analysis (RDA; Fig. 4) concurs with the results obtained by the questionnaire, suggesting that the mean abundance of *G. cichlidarum*, *Trichodina* sp. and *C. halli* in medium- to high-technology farms was positively statistically associated with high fish densities per tank, a large number of workers, the lack of a quarantine area and high concentrations of nitrites. The farms where production was highest may be raising fish at higher densities and could be employing proportionately more staff than the farms where production is lower [46]. However, even under these more favourable circumstances, these farms are still infected with ectoparasites due to the lack of technical training of the farmers.

The second group of farms (low-technology farms), where the Nile tilapia were infected with several species of *Cichlidogyrus*, also had deficient management practices and substandard sanitary conditions such as a lack of preventive treatments, insufficient disinfection equipment and tanks, poor fingerling sources, and deficient water quality (Table 3). The mean abundance of these ectoparasites was also positively associated with high ammonium concentrations and a deficient water exchange rate. Farms in this group were technologically disadvantaged compared to the first farm group. The owners’ lack of awareness in this farm group concerning fish disease or the adverse effects of disease outbreaks is reflected in the poor preventive and treatment measures for eliminating ectoparasites in the fingerlings before they are introduced into the production areas. Several farmers used chemical products such as organophosphates for eliminating parasites in the fingerlings but had little understanding of their use, effectiveness and ecological consequences (Table 3). Better results might have been obtained by improved management practices such as the use of salt baths because of their low cost and effectiveness against bacteria, viruses and parasites [47, 48]. It is highly probable that most farms from both groups have a few main suppliers of infected fingerlings, which likely serve as the most serious threat of ectoparasite transmission. This situation, coupled with the deficient management conditions of each Nile tilapia farm, is reflected in the high parasite prevalence. Faruk et al. [49] found that small rural farms in Bangladesh had a high prevalence of diseases such as white spot, epizootic ulcerative syndrome and red spot, when compared to larger, highly technological farms. This situation can be explained because low-technology farms lack proper assistance from the government, medicine is unavailable, and there is a lack of training and facilities for the treatment of fish diseases. Nile tilapia fingerlings should be treated prior to being transported to farms; however, to apply such treatments, a sensitivity analysis needs to be undertaken to determine the appropriate concentrations of chemicals for killing the parasite without affecting the fish [47]. In addition, proper quarantine programs need to be established prior to introducing fingerlings into a farm. Wagner et al. [50] found that farms in the United States (Alabama, Arkansas, Louisiana, and Mississippi) that did not produce their own fingerlings were more likely to report enteric septicemia problems compared to those that produced their own fingerlings*.* A reason for this phenomenon could be that the sanitary conditions of the farms that produce their own fingerlings would be well known, and stocking decisions would then be based on that knowledge. In addition, handling or transporting can produce stress in fish, as well as stocking outside appropriate temperature ranges, causing immunosuppression conducive to disease outbreaks [51]. Because of the association between deficient management practices and the increase in ectoparasitic infections, technical training programs are needed to improve the management practices of both workers and owners of the Nile tilapia farms in Yucatán.

### Effect of management and environmental variables on the mean abundance of *Gyrodactylus cichlidarum*

The General Additive Model for Location Scale and Shape (GAMLSS; Table 4) suggested that the use of a quarantine area and prophylactic treatments, together with low values of dissolved oxygen (5–8 mg/l) in large tanks (12–14 m^**3**^), increase the mean abundance of *G. cichlidarum* for high-tech farms. Thus, in those farms, the newcomers (fry or broodstock) are not being appropriately isolated in specific quarantine facilities, examined for parasites and, when necessary, treated before their release into the production areas. The absence of the adequate application of these management procedures clearly increases the probability of the transmission and reproduction of *G. cichlidarum* not only in the newcomers but also in the Nile tilapia already present in the farms. To the best our knowledge, there are no safe concentrations for sodium chloride or other substances for therapeutic control of *G. cichlidarum*. Clearly, the most effective concentrations for these substances for killing the parasite without killing the fish need to be determined [47]. In cultured Nile tilapia, outbreaks of gyrodactylosis have been suggested as being responsible for the high mortality of juvenile fish in a number of countries worldwide [52]. Studies in temperate latitudes have reported that risk factors such as the reuse of the equipment (especially hand nets without adequate disinfection) and the movement of farm equipment among tanks have caused the spread of *Gyrodactylus salaris* Malmberg, 1957 between salmon farms in Norway [45, 52]. Therefore, good sanitary practices together with proper therapeutic treatments are necessary to control the dispersion and establishment of *G. cichlidarum* within and between Nile tilapia farms in Yucatán.

## Conclusion

The Nile tilapia farms in the state of Yucatán generally have substandard management conditions that play an important role in the high occurrence of parasitic infections. Both the geographical and multivariate analyses sought to identify high-risk locations and factors that might be associated with risky practices. Several of these practices were related to the distribution of infected fingerlings among farms, especially in the northwestern corner of Yucatán, the region where the most frequent and abundant parasites showed significant clusters. However, something that both high- and low-technology farms shared was the urgent need for proper technical training programs to improve the management and sanitary practices of both workers and owners of the Nile tilapia farms. We consider Yucatán a good model when trying to understand the sanitary circumstances of Nile tilapia farming in a developing country, with the results obtained here applying to rural areas of most countries in the Neotropics. We certainly acknowledge the consideration of FAO with respect to the fast growth of aquaculture worldwide [1], but if the benefit of this growth is to reach people living in the rural regions of these Neotropical countries, proper technical training for farmers is urgently needed to meet this expectation.

## Additional file

Additional file 1:
**Answers from the owners of the 21 Nile tilapia farms in Yucatán (the ones that were willing to cooperate) to the questionnaire on their management practices and sanitary measures.** (TIF 1660 kb) Additional file 2:
**Clustered patterns of the parasitic helminths and protozoans of the Nile tilapia** ***Oreochromis niloticus*** (L.) in Yucatán. Values of Ripley’s *K* as a function of the radius (r) of the kernels from the point patterns calculated for each one of the ectoparasite species of the Nile tilapia for all 29 extant farms in Yucatán, México. If the observed Ripley´s *K* function (K_obs_) was above the theoretical Ripley´s *K* function (K_theo_), thus the point pattern was considered as non random. If the point pattern of one species fulfilled the assumption of non randomness, then its intensity (mean abundance) was estimated by a nonparametric method, and kernel smoothing using an isotropic Gaussian kernel was carried out. K_hi_ and K_low_ are the confidence intervals. (PDF 89 kb) Additional file 3:
**Generalized additive model functions of** ***Gyrodactylus cichlidarum*** **Paperna, 1968 mean abundance (dependent variable) in relation to independent management and environmental variables (quarantine area, prophylactic treatments, dissolved oxygen, tank capacity).** The dotted line along the X axis indicates the 95 % confidence intervals for each explanatory variable. The partial residuals of the constant model for each variable are plotted along the Y axis. (TIF 34 kb)
