# Supplementary figures and images for: Geographical distribution of protozoan and metazoan parasites of farmed Nile tilapia Oreochromis niloticus (L.) (Perciformes: Cichlidae) in Yucatán, México
Source: Parasit Vectors. 2016 Feb 3;9:66. doi: 10.1186/s13071-016-1332-9 (PMC4740987; doi:10.1186/s13071-016-1332-9)

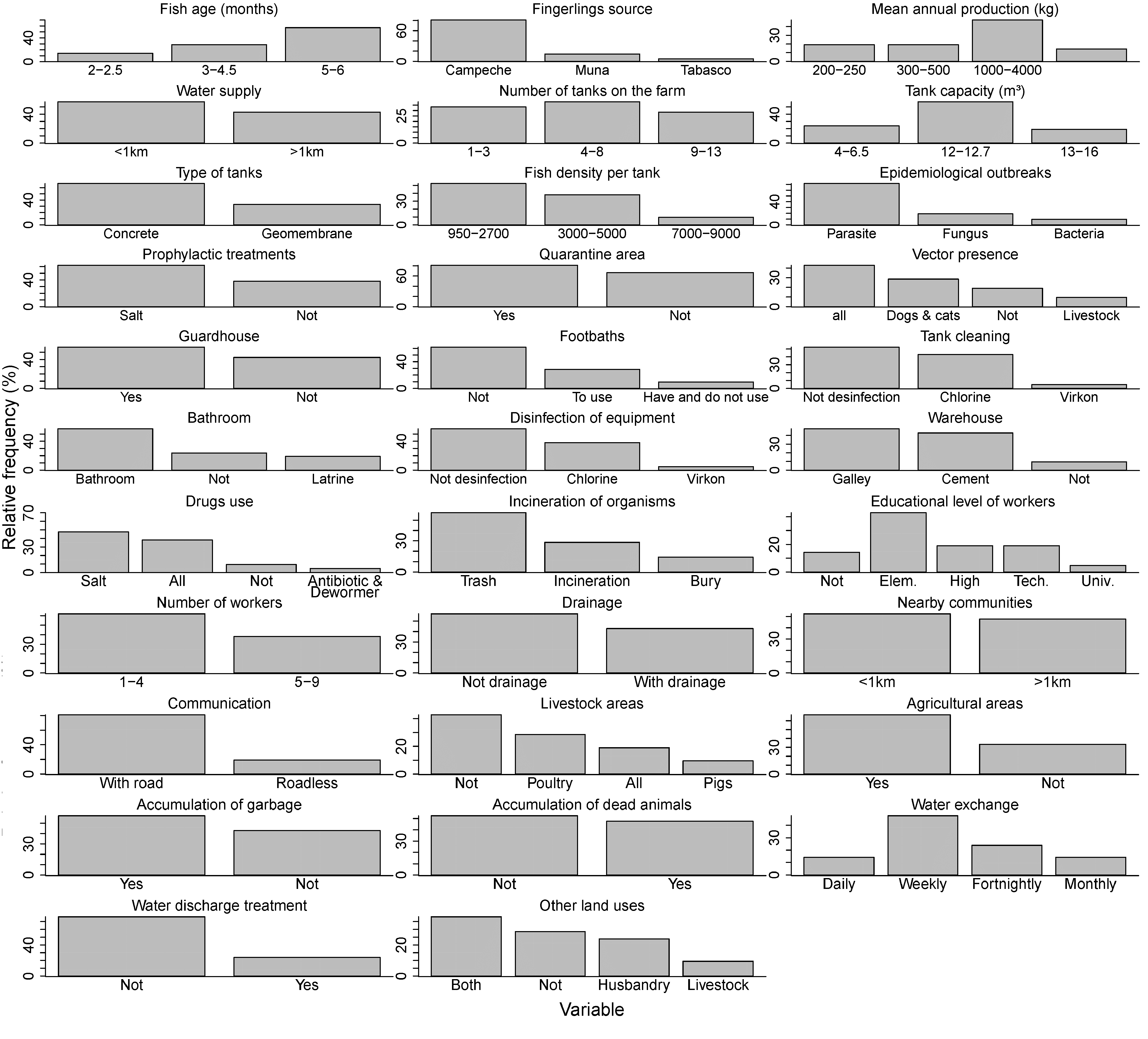

Supplement: Additional file 1: — Answers from the owners of the 21 Nile tilapia farms in Yucatán (the ones that were willing to cooperate) to the questionnaire on their management practices and sanitary measures. (TIF 1660 kb) [file 13071_2016_1332_MOESM1_ESM.tif]
