# Supplementary material for: Geographical distribution of protozoan and metazoan parasites of farmed Nile tilapia Oreochromis niloticus (L.) (Perciformes: Cichlidae) in Yucatán, México
Source: Parasit Vectors. 2016 Feb 3;9:66. doi: 10.1186/s13071-016-1332-9 (PMC4740987; doi:10.1186/s13071-016-1332-9)
Supplement: Additional file 2: — Clustered patterns of the parasitic helminths and protozoans of the Nile tilapia Oreochromis niloticus (L.) in Yucatán. Values of Ripley’s K as a function of the radius (r) of the kernels from the point patterns calculated for each one of the ectoparasite species of the Nile tilapia for all 29 extant farms in Yucatán, México. If the observed Ripley´s K function (Kobs) was above the theoretical Ripley´s K function (Ktheo), thus the point pattern was considered as non random. If the point pattern of one species fulfilled the assumption of non randomness, then its intensity (mean abundance) was estimated by a nonparametric method, and kernel smoothing using an isotropic Gaussian kernel was carried out. Khi and Klow are the confidence intervals. (PDF 89 kb) [file 13071_2016_1332_MOESM2_ESM.pdf]

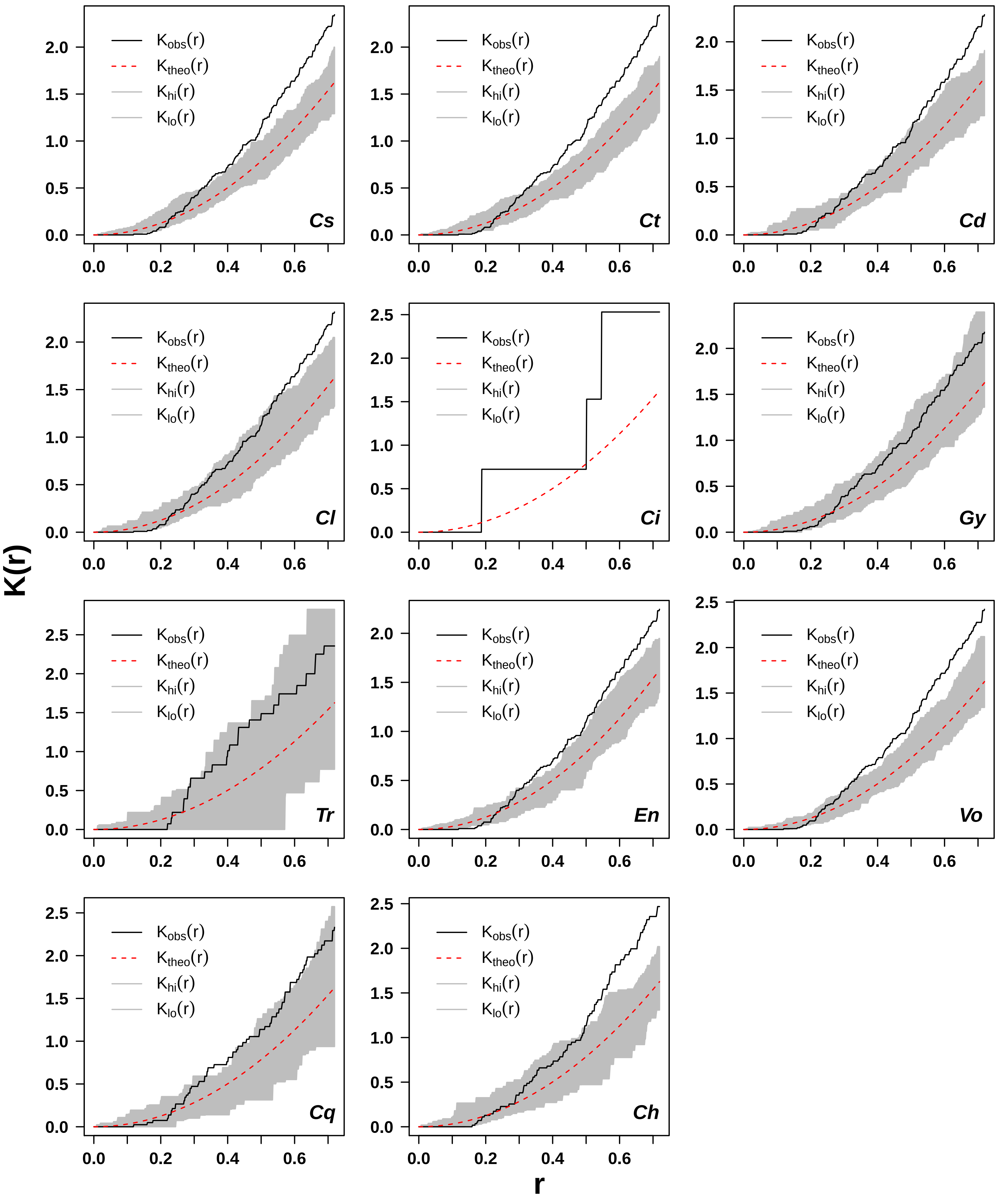
K(r)
r
Kobs(r)
Ktheo(r)
Khi(r)
Klo(r)
Cs
Ct
Cd
Cl
Ci
Gy
Tr
En
Vo
Cq
Ch
